# Factors Correlated to the Renoprotective Effect of Sitagliptin in Patients with Type 2 Diabetes Mellitus: Retrospective Observational Study

**DOI:** 10.1155/2024/7181515

**Published:** 2024-08-30

**Authors:** Pirawan Khunkit, Konkanok Wattana

**Affiliations:** ^1^ Department of Pharmaceutical Care Walailak University, Tha Sala, Nakhon Si Thammarat 80160, Thailand; ^2^ Drug and Cosmetics Excellence Center Walailak University, Tha Sala, Nakhon Si Thammarat 80161, Thailand

## Abstract

**Background:**

Sitagliptin functions similarly to GLP-1RAs by incretin and insulin secretion and has a renoprotective effect. Diabetic kidney disease (DKD) is a kidney complication that increases the mortality rate in type 2 diabetes mellitus (T2DM) patients. The important parameters that predict appropriate sitagliptin treatment are known as factors. This study aimed to assess factors that correlated with the renoprotective effect of sitagliptin in patients with T2DM.

**Methods:**

This retrospective study collected data from a tertiary hospital in Thailand. All T2DM patients who were treated with sitagliptin and had complete data were recruited to analyze the outcome. The primary outcome was a correlation between demographics, laboratory data, and kidney outcome. The secondary outcome was the different laboratory results between pre- and posttreatment of patients treated with sitagliptin.

**Results:**

The number of patients who were treated for T2DM with sitagliptin was 191. Only 102 patients had complete laboratory parameters. Results showed a positive correlation between baseline FBS, Hb_A1c_, and Scr change (*p* value = 0.042 and 0.005) at 6 months and baseline age, TG, and Scr change (*p* value = 0.010 and 0.022) at 18 months; while a negative correlation was observed between baseline FBS, Hb_A1c_, and eGFR change (*p* value = 0.017 and 0.007) at 6 months and baseline age and eGFR change (*p* value = 0.010) and between HDL-cholesterol and Scr change at 18 months (*p* value = 0.044). The eGFR stage 1 subgroup showed a positive correlation between baseline Hb_A1c_ and Scr change (*p* value <0.001) and baseline DM duration and eGFR change (*p* value = 0.004). Moreover, sitagliptin showed statistically significant FBS, Hb_A1c_, LDL-cholesterol, and TC reduction. Furthermore, HDL-cholesterol showed statistically significant elevation.

**Conclusion:**

FBS, HbA1c, and age were factors that correlated with the renoprotective effect of sitagliptin. The eGFR ≥90.00 ml/min/1.73 m^2^ patients group showed a duration of DM in which factors correlated with renoprotective effect. Moreover, sitagliptin also can improve glucose levels and lipid profile.

## 1. Introduction

Type 2 diabetes mellitus (T2DM) is one of the noncommunicable diseases (NCDs) that cause insulin resistance. Most of the patients are adults [1]. T2DM patients who cannot have blood glucose level control for a long time lead to many complications, especially chronic kidney disease (diabetic kidney disease (DKD)). DKD is one of the microvascular complications that can be found after a diabetes duration of 10 years in type 1 diabetes [2]. On the other hand, T2DM is still unclear. The time to development of DKD depends on risk factors such as type of diabetes, age at diagnosis, glucose level, blood pressure level, lipid level, alcohol use, and obesity [3, 4]. This phenomenon was diagnosed by albuminuria and/or reduced estimated glomerular filtration rate (eGFR) in patients with the absence of signs or symptoms of other causes of kidney damage. Generally, eGFR is reported with serum creatinine in the routine laboratory. In addition, it can be calculated from the Chronic Kidney Disease Epidemiology Collaboration (CKD-EPI) equation by using serum creatinine in the formula. So, DKD also can be indicated via serum creatinine change. 20–40% of adults with chronic kidney disease were reported to have diabetic kidney disease. Moreover, this complication is a high risk to mortality rate. The previous study demonstrated that incidence rates per 1000 person-years in DKD patients reported 6.9 for end-stage renal disease (ESRD) and 50.3 for mortality [5]. Therefore, T2DM patients should be screened for kidney function at least annually to assess kidney function [2]. American Diabetes Association recommended sodium-glucose cotransporter 2 inhibitors (SGLT2is) and glucagon-like peptide 1 receptor agonists (GLP-1RAs) for T2DM with chronic kidney disease (CKD) or high risk of chronic kidney disease treatment [2, 6].

Sitagliptin is one of the antihyperglycemic agents in the dipeptidyl peptidase 4 inhibitors (DPP4is) class. This agent acts to inhibit the degradation of the incretin hormones similar to GLP-1RAs [7]. On the other hand, this class is rarely mentioned for T2DM patients with high-risk DKD or renal impairment treatment. DPP4is class consists of vildagliptin, linagliptin, alogliptin, and saxagliptin, including sitagliptin. The linagliptin study reported risk reduction and slowed kidney function progression [8], whereas no clinically significant changes in renal function were reported [9–11]. In addition, T2DM patients who were treated with saxagliptin showed good tolerance also in moderate and severe kidney disease [12, 13]. For vildagliptin, the previous study demonstrated safety in moderate and severe CKD with T2DM patients [14, 15]. In addition, some studies showed an increase in blood urea nitrogen (BUN) slightly, but it was not statistically significant [16]. Furthermore, alogliptin was administered in patients with moderately and severely impaired renal function or ESRD. The study reported a change in eGFR according to the baseline kidney function and incidences of initiation of dialysis were not different between the alogliptin and placebo groups [17]. Almost all these agents' studies showed a renoprotective effect. For sitagliptin, the previous study demonstrated that sitagliptin affects eGFR reduction in each eGFR stage group [18]. The study by Chan et al. reported a decrease in serum creatinine for patients with moderate renal insufficiency [19]. However, the sitagliptin studies about kidney outcomes in humans are fewer when compared to the other agents in this class.

As mentioned above, the mechanism of action and the previous study of sitagliptin showed the renoprotective effect. Moreover, DKD is an important complication that increases the mortality rate in T2DM patients. This complication is the absence of signs or symptoms until it develops into ESRD or proceeds to the renal failure stage. Some patients need dialysis or transplantation. Nowadays, there is no report of factors for identifying T2DM patients who should receive sitagliptin approximately to slow DKD progression and renal failure. Therefore, this study aimed to investigate the factors correlated with the renoprotective effect of sitagliptin in patients with T2DM. These factors are beneficial to planning sitagliptin prescriptions.

## 2. Materials and Methods

### 2.1. Patient Population

This study's design is a retrospective observational study. All data were screened from a tertiary hospital in Thailand. The inclusion criteria were patients who were older than 18 years, were diagnosed with type 2 diabetes mellitus (T2DM), and were prescribed sitagliptin as one of the T2DM treatment regimens. They were excluded if they were treated with SGLT2is, GLP-1RAs, and other DPP4is. In addition, they also were excluded if laboratory data were not complete, i.e., there was no kidney laboratory result (eGFR and Scr) before treatment and there was a kidney laboratory result from only one visit.

For patients who were recruited in this retrospective study, we collected information and laboratory data such as age, body weight, height, comorbid diseases, concurrent medication, diabetes mellitus duration, eGFR, Scr, systolic blood pressure (SBP), diastolic blood pressure (DBP), fasting blood sugar (FBS), hemoglobin A1c (Hb_A1c_), low-density lipoprotein cholesterol (LDL-cholesterol), high-density lipoprotein cholesterol (HDL-cholesterol), triglyceride (TG), and total cholesterol. This information was recorded at the first visit when patients were prescribed sitagliptin as baseline parameters. In addition, these parameters were collected at the 6-month, 12-month, and 18-month visits.

### 2.2. Outcome Measurement

The primary outcome was the correlation between kidney laboratory results, i.e., serum creatinine (Scr) and estimated glomerular filtration rate (eGFR) change and other variables, such as age, body mass index (BMI), diabetes mellitus duration, systolic blood pressure (SBP), diastolic blood pressure (DBP), fasting blood sugar (FBS), hemoglobin A1c (Hb_A1c_), total cholesterol, triglyceride, HDL-cholesterol, LDL-cholesterol, aspartate transaminase (AST), and alanine transferase (ALT) of the T2DM patients treated with sitagliptin. The correlation shows the factors that affect kidney complications in people with diabetes mellitus. Secondary outcomes were different in Scr, eGFR, BMI, FBS, Hb_A1c_, total cholesterol, triglyceride, HDL-cholesterol, and LDL-cholesterol between pre- and posttreatment of T2DM patients treated with sitagliptin.

Renoprotection was defined as a phenomenon that interrupts or reverses renal function loss. It was indicated via renal parameters, i.e., eGFR, Scr, or albumin level in urine [20, 21].

### 2.3. Statistical Analysis

Baseline characteristics on demographic data of all patients were reported as numbers and percentages. Baseline laboratory data were reported as median and 95% confidence interval (95% CI). Linear regression was used to investigate the association between the factors and kidney function parameters. The correlation between serum creatinine (Scr) and estimated glomerular filtration rate (eGFR) change and expected relevant variables was performed using Spearman correlation analysis. Comparison of Scr, eGFR, BMI, FBS, HbA1c, total cholesterol, triglyceride, HDL-cholesterol, and LDL-cholesterol between pre-and posttreatment of sitagliptin was performed using the Wilcoxon signed-rank test. All statistical analyses were performed with IBM SPSS Statistics software version 28.0.0.0 (190). Statistical significance was set as *p* value <0.05, and power was 80%.

### 2.4. Ethical Approval

The methodology of this study was approved by the Human Research Ethics Committee of Walailak University (HREC WU; registration no. WUEC-24-030-01). The requirement of patient informed consent was waived because it was a retrospective study that did not directly involve any patient. All patient data were collected and processed following the Declaration of Helsinki.

## 3. Results

### 3.1. Baseline Characteristics

**Figure 1:**
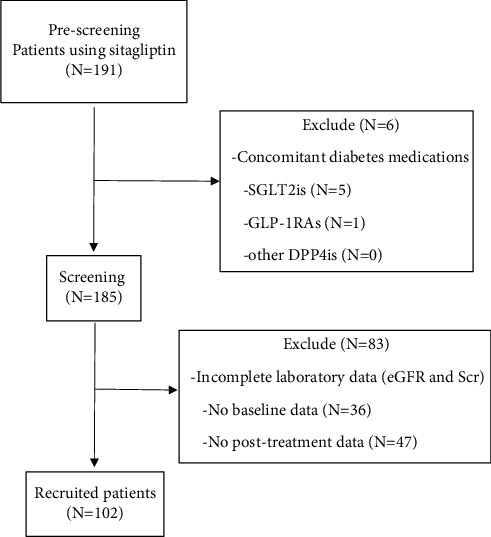
Flow diagram of the patient recruitment process.

The baseline characteristics are shown in Table 1. The number of patients who were treated for type 2 diabetes mellitus (T2DM) from January 2021 to November 2023 with sitagliptin was 191. 102 patients had complete laboratory parameters. So, these patients were analyzed in this study (Figure 1). The number of males and females was similar. The majority of these patients were younger than 60 years old (44.12%). Markedly, patients had body mass index (BMI) higher than 25.00 kg/m^2^ (60.78%) and the most common comorbidity diseases of patients were hypertension and dyslipidemia. The number of patients with each disease was equal (56.86%). Concomitant medications especially diabetes medications showed biguanides (87.25%), sulfonylureas (41.18%), insulin (18.63%), and thiazolidinediones (9.80%). For renin-angiotensin system (RAS) inhibitors, medications were 47.06%, i.e., angiotensin receptor blockers (ARBs) (30.39%) and angiotensin-converting enzyme inhibitors (ACEis) (16.67%). Furthermore, T2DM duration, i.e., less than 10, 11–20, 21–30, and more than 30 years was 52.94, 31.37, 11.76, and 3.92%, respectively. The number of patients' smoking status in each group was almost the same. The CVD risk score that was calculated from the atherosclerotic cardiovascular disease (ASCVD) risk estimator plus calculator [22] showed the number of patients over half in less than 10% group. For median baseline kidney parameters, patients reported that the estimated glomerular filtration rate (eGFR) was 90.80 ml/min/1.73 m^2^ and serum creatinine (Scr) was 0.84 mg/dL. Most patients showed baseline eGFR in eGFR stage 1 (≥90.00) which was classified from chronic kidney disease classification of Kidney Disease Improving Global Outcomes (KDIGOs) 2024 [23] (49.02%). In addition, baseline eGFR of individual patients showed eGFR stage 2, stage 4, stage 3, and stage 5 (37.25, 6.86, 5.88, and 0.98, respectively). Baseline proteinuria was classified by the sulfosalicylic acid test (SSA) that was graded from turbidity urine [24]. Almost all patients showed negative results (93.14%). For the median of baseline, blood pressure showed normal levels. The median of fasting blood sugar (FBS), hemoglobin A1c (Hb_A1C_), total cholesterol, triglyceride, HDL-cholesterol, LDL-cholesterol, aspartate transaminase (AST), and alanine transferase (ALT) was 184.00 mg/dL, 8.70%, 186.00 mg/dL, 148.00 mg/dL, 51.00 mg/dL, 107.00 mg/dL, 20.00 IU, and 21.00 IU, respectively.

### 3.2. Effect on Kidney and Cardiometabolic Parameter

**Figure 2:**
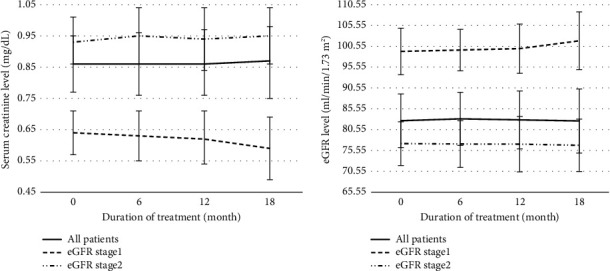
Differences in serum creatinine levels in all patients, patients who have estimated glomerular filtration rate (eGFR) stage 1, and patients who have eGFR stage 2, (a) differences in eGFR levels in all patients, patients who have eGFR stage 1, and patients who have eGFR stage 2 (b) between 6-month, 12-month, and 18-month posttreatment compared with pretreatment; bar chart presents mean and 95% confidence interval.

Prior to the sitagliptin treatment, this study showed that the mean of serum creatinine (Scr) was 0.86 mg/dL. At 6, 12, and 18-month duration of sitagliptin treatment, the mean of Scr was 0.86, 0.86, and 0.87 mg/dL, respectively. This study showed no statistically significant difference (*p* value = 0.497, 0.499, and 0.484, respectively) between pre- and posttreatment at 6, 12, and 18 months. Moreover, subgroup patients who have baseline estimated glomerular filtration rate (eGFR) of ≥90.00 ml/min/1.73 m^2^ (eGFR stage 1) and eGFR 60.00–89.99 ml/min/1.73 m^2^ (eGFR stage 2) showed a mean of Scr in the pre- and posttreatment at 6, 12, and 18 months; eGFR stage 1: 0.64, 0.63, 0.62, and 0.59 mg/dL; and eGFR stage 2: 0.93, 0.95, 0.94, and 0.95 mg/dL, respectively. Similarly, differences in Scr between pre- and posttreatment at 6, 12, and 18 months showed no statistically significant difference for eGFR stage 1 and 2 groups (eGFR stage 1: *p* value = 0.453, 0.718, and 0.116; eGFR stage 2: *p* value = 0.170, 0.475, and 0.313 respectively) (Figure 2(a)). The effect on the eGFR parameter is shown in Figure 2(b). This study compared the difference between pre- and posttreatment at 6, 12, and 18 months in all patients, eGFR stage 1, and eGFR stage 2 groups. Mean eGFR pre- and posttreatment for all patients were 82.69, 83.13, 82.88, and 82.63 ml/min/1.73 m2; eGFR stage 1: 99.31, 99.63, 99.97, and 101.86; and eGFR stage 2: 77.19, 77.09, 77.07, and 76.78, respectively. This study showed no statistically significant difference (all patients: *p* value = 0.178, 0.872, and 0.276; eGFR stage 1: *p* value = 0.176, 0.614, and 0.508; and eGFR stage 2: *p* value = 0.153, 0.713, 0.251, respectively) between pre- and posttreatment in all groups. The effects on proteinuria posttreatment at 6, 12, and 18 months in all patients showed 4, 6, and 8 patients; in eGFR stage 1 group, there were 1, 1, and 5 patients; and in eGFR stage 2 group, there were 3, 4, and 3 patients, respectively. This study showed a statistically significant difference in the number of proteinuria patients between posttreatment at 6 and 18 months in all patients and 6 and 18 months and 12 and 18 months in the eGFR stage 1 group (all patients: *p* value = 0.019 and eGFR stage 1: *p* value = 0.005 and 0.018, respectively).

**Figure 3:**
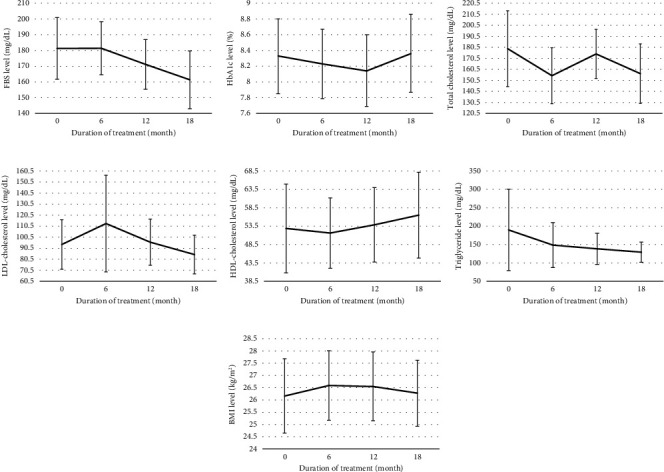
Differences in FBS (a), HbA1c (b), total cholesterol (c), LDL-cholesterol (d), HDL-cholesterol (e), triglyceride (f), and BMI (g) between 6-month, 12-month, and 18-month posttreatment compared with pretreatment; bar chart presents mean and 95% confidence interval.

In addition, the effect of sitagliptin on cardiometabolic parameters showed mean of FBS, Hb_A1c_, total cholesterol (TC), LDL-cholesterol, HDL-cholesterol, triglyceride (TG), and BMI in pre- and posttreatment at 6, 12, and 18 months (FBS: 184.85, 186.90, 172.72, and 165.20 mg/dL; Hb_A1c_: 8.40, 8.39, 8.30, and 8.48%; TC: 167.67, 150.87, 168.33, and 156.93 mg/dL; LDL-cholesterol: 117.24, 100.97, 101.21, and 101.06 mg/dL; HDL-cholesterol: 53.87, 54.00, 57.40, and 57.27 mg/dL; TG: 165.60, 133.33, 138.80, and 133.67 mg/dL; and BMI: 26.06, 26.25, 26.23, and 26.03 mg/dL, respectively). The result of this study reported statistically significant FBS and LDL-cholesterol reduction in posttreatment at 6, 12, and 18 months (FBS: *p* value = 0.040, 0.008, and 0.004 and LDL-cholesterol: *p* value <0.001, = 0.002, and 0.020, respectively) (Figures 3(a) and 3(d)). On the other hand, TG and BMI reported no statistically significant differences between pre- and posttreatment at 6, 12, and 18 months (Figures 3(f) and 3(g)). For Hb_A1c_ and TC, a statistically significant reduction in posttreatment at 6 months was observed (*p* value = 0.013 and 0.001, respectively) (Figures 3(b) and 3(c)). Furthermore, HDL-cholesterol showed a statistically significant increase in posttreatment at 12 months (*p* value = 0.005) (Figure 3(e)).

### 3.3. Factors Correlated to the Renoprotective Effect

The result of this study demonstrated no statistically significant difference for eGFR and Scr between pre- and postsitagliptin treatment in all patients, eGFR stage 1, and eGFR stage 2 groups. Almost all patients reported normal eGFR and Scr levels at baseline. It means sitagliptin can slow the progress of chronic kidney disease or diabetic kidney disease (DKD) complications. Therefore sitagliptin has a renoprotective effect.  Table 2 shows factors that correlate with kidney outcomes change in T2DM patients. At 6 months of sitagliptin treatment, there was a positive correlation between baseline FBS, Hb_A1c_, and Scr change. However, there was a negative correlation between baseline FBS, Hb_A1c_, and eGFR change. It indicated that high baseline FBS and Hb_A1c_ correlate with Scr elevation highly (*p* value = 0.042 and 0.005) and eGFR reduction slowly (*p* value = 0.017 and 0.007). Moreover, regression analysis indicated that 0.88% of Hb_A1c_ elevation affected 1 ml/min/1.73 m^2^ of eGFR decreasing and 0.02% of Hb_A1c_ elevation affected 1 mg/dL of Scr increasing. At 18 months of sitagliptin treatment, there was a positive correlation between baseline age, TG, and Scr change, whereas there was a negative correlation between baseline age and eGFR change. It indicated that high baseline age and TG correlate with Scr elevation highly (*p* value = 0.010 and 0.022). At the same time, high baseline age also correlates with eGFR reduction slowly (*p* value = 0.010). Moreover, the result also showed a negative correlation between HDL-cholesterol and Scr change. It means high baseline HDL-cholesterol levels correlate with Scr elevation slowly (*p* value = 0.044). Regression analysis indicated that 1 year of age elevation affected 1 ml/min/1.73 m^2^ of eGFR decreasing.

**Figure 4:**
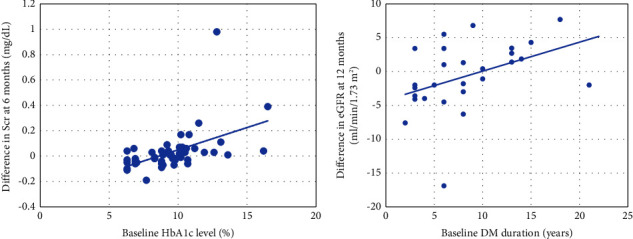
Correlation between baseline hemoglobin A1c (Hb_A1c_) (a) and difference in serum creatinine (Scr) of each patient who has eGFR stage 1 treated with sitagliptin for 6 months and baseline diabetes mellitus (DM) duration and difference in estimated glomerular filtration rate (eGFR) (b) of each patient who has eGFR stage 1 treated with sitagliptin for 12 months.

Focusing on the baseline eGFR subgroup, the result of this study demonstrated a positive correlation between baseline Hb_A1c_ and Scr change in T2DM patients in eGFR stage 1. It indicated that high baseline Hb_A1c_ correlates with Scr elevation highly (*p* value <0.001) (Figure 4(a)). Regression analysis indicated that 0.05% of Hb_A1c_ elevation affected 1 mg/dL of Scr increasing. Moreover, the result also showed a positive correlation between baseline DM duration and eGFR change. It means high baseline DM duration correlates with eGFR reduction highly (*p* value = 0.004) (Figure 4(b)).

## 4. Discussion

The result of this study reported no statistically significant difference in eGFR and Scr between pre- and postsitagliptin treatment. It indicated that sitagliptin can slow the progress of chronic kidney disease or diabetic kidney disease (DKD) complications. Markedly, sitagliptin was the renoprotective antihyperglycemic agent. For albuminuria, we tried our best to ensure albuminuria. Since the limitation of the retrospective study, a parameter that indicated albuminuria may not be clear. However, this study indicated that sitagliptin affected proteinuria elevation posttreatment at 18 months. The previous study reported that sitagliptin reduced albuminuria via blood glucose control [25–28]. Moreover, preliminary data reported that albuminuria change was lower than the placebo group [29]. We suggest collecting albumin levels in urine for prospective study in future. The previous study reported no change in eGFR rate in patients not on hemodialysis [30]. Moreover, ten-year sitagliptin treatment also reported no change in eGFR [31]. DPP4 enzyme showed the highest expression levels in mammals' kidneys [32]. Consequently, the kidney is an important target organ for sitagliptin. Incretin has been known for kidney function regulation [33, 34], whereas T2DM patients showed incretin dynamics alteration, i.e., vascular tonus, diuretic, and natriuretic properties in kidney and DPP4 upregulation in glomeruli for T2DM patients. In addition, DPP4 also involved extracellular matrix proteins during DKD development and vascular endothelial growth factor (VEGF) regulation which have key roles in the pathophysiology of DKD [35]. Therefore incretin hormone (GLP-1) in the kidney may have a half-life reduction. [32, 36, 37]. The aforementioned result of this study is the renoprotective effect of sitagliptin as a result of DPP4 inhibition. Since the DKD stage was classified by eGFR alteration and albuminuria [38], therefore, different DKD stages have different effects on DPP4 inhibitors.

Interestingly, this study indicated that T2DM patients with high baseline FBS or Hb_A1c_ correlated with eGFR reduction slowly at 6 months of sitagliptin therapy. Moreover, sitagliptin can reduce FBS and Hb_A1C_ levels. Similarly, the previous study demonstrated that sitagliptin can initially reduce Hb_A1c_ levels in 24 weeks. T2DM patients with higher baseline Hb_A1c_ lead to greater reductions in Hb_A1c_ compared with the lower baseline Hb_A1c_ group [39]. Sitagliptin also decreases FBS levels, and there is no significant difference when compared to pioglitazone [40]. Since the agent inhibits the DPP4 enzyme that cleavages GLP-1 and glucose-dependent insulinotropic polypeptide (GIP), this mechanism increases insulin and reduces glucagon. It leads to improved T2DM [41, 42]. Effect of sitagliptin on high Hb_A1c_ level reduction in high baseline Hb_A1c_ patient affect slow DKD progression in these patients. High blood glucose for a long time can induce oxidative stress and proinflammatory cytokines release which is the cause of DKD [43–45]. In addition, sitagliptin was reported to prevent proapoptotic and inflammatory states affecting renal function improvement in diabetic rat kidneys [46]. On the other hand, these factors affected Scr elevation highly. Creatinine is a metabolite of creatine and creatine phosphate which is a protein in skeletal muscle [47]. Muscle mass and dietary meat intake have been shown to influence Scr levels [5, 48, 49]. High FBS and Hb_A1c_ levels affect muscle mass change. However, GFR is the gold standard of CKD diagnosis [50]. GFR is estimated by creatinine clearance (Clcr) that is measured by Scr and creatinine excretion in a 24-h urine sample. Generally, eGFR equations consist of many parameters, i.e., age and gender, including Scr [51]. Therefore, GFR is the best parameter for kidney function assessment in health and disease [52]. Consequently, we focus on eGFR change for the renoprotective effect. By focusing on subgroups via baseline eGFR, the result of this study showed a positive correlation between baseline DM duration and eGFR reduction in the eGFR stage 1 group; eGFR ≥90.00 ml/min/1.73 m^2^. T2DM patients who were affected by DM for a long time at baseline correlated with eGFR reduction highly at 12 months. A previous study demonstrated that the average duration of T2DM was 5–10 years, which is related with the severity of nephropathy [53]. Notably in this study, the majority of DM duration in the eGFR stage 1 group was less than 10 years. This phenomenon indicated that sitagliptin can slow the progress of DKD via DM control and inflamed process control, especially in T2DM patients who had uncontrol DM and were in older age. However, in patients with baseline eGFR of ≥90.00 ml/min/1.73 m^2^ who had T2DM for a long time, sitagliptin monotherapy was not recommended. These patients should receive treatment combined with other antihyperglycemic agents such as SGLT2is or GLP-1RAs to slow DKD progression.

Moreover, the current study reported that high baseline age patients correlated with eGFR reduction slowly at 18 months. It is known that the elderly population has declined in eGFR [23, 54]. This phenomenon is possible because besides sitagliptin having benefits for glucose level control, the agent can control metabolic parameters. Results of this study showed that sitagliptin decreases LDL-cholesterol and total cholesterol levels with at least 6 months of treatment. At 12 months, HDL-cholesterol showed elevation. Previous studies in rabbits showed that sitagliptin decreased LDL-cholesterol and total cholesterol including reversing the decrease in HDL-cholesterol levels [55]. Furthermore, the human study reported that sitagliptin improves total cholesterol, HDL-cholesterol, LDL-cholesterol, and TG levels [56], while the present study showed no significant decrease in the TG level. However, the effect of other DPP4is agents on metabolic parameters change is different [57, 58]. This result was indicated by the impact of incretin on lipid metabolic enzymes. It affects lipogenesis and lipolysis [59]. Moreover, a previous study reported that sitagliptin decreased lipid peroxidation. It may improve renal impairment [28]. The capacity of sitagliptin on glucose and lipid control affects to improve of DM and dyslipidemia which is a comorbidity disease of almost all patients in this study and a risk factor for DKD development [60]. Generally, older patients are at high risk for these diseases [61]. Therefore it can be concluded that high baseline age patients should receive sitagliptin to slow DKD progression. In addition, sitagliptin treatment demonstrated no statistically significance difference in BMI. The previous study reported a weight-neutral effect [62]. Since BMI is a parameter that is calculated via weight and height [63], so weight change can affect BMI.

To the best of our knowledge, the current study demonstrated that baseline FBS, Hb_A1c_, and age were factors that correlated with the renoprotective effect of sitagliptin. Therefore, T2DM patients with high baseline FBS, HbA1c, and older age should receive sitagliptin to slow DKD progression. For patients with T2DM and a baseline eGFR ≥90.00 ml/min/1.73 m2, who have had diabetes for a prolonged period, it is recommended to consider treatment with sitagliptin combined with SGLT2is or GLP-1RAs to achieve enhanced renoprotective benefits. Unfortunately, the limitation of this retrospective study is the number of patients who have complete data including pre- and postsitagliptin treatment and parameter collection that indicated albuminuria. It also cannot indicate the cutoff level of age and DM duration as a high group, including urine albumin level, which may be a factor for this study. Further studies should collect data from a higher number of samples for more clarity.

## 5. Conclusion

The result of this study indicates that sitagliptin is a renoprotective antihyperglycemic agent. FBS, HbA1c, and age were factors that correlated with the renoprotective effect of sitagliptin. Moreover, the duration of DM was a factor that correlated with the renoprotective effect of sitagliptin for T2DM patients with eGFR ≥90.00 ml/min/1.73 m^2^. Sitagliptin also can improve glucose levels and lipid profile, i.e., FBS, Hb_A1c_, LDL-cholesterol, HDL-cholesterol, and total cholesterol. Based on the results of this study, baseline FBS, Hb_A1c_, age, and DM duration should be considered for sitagliptin treatment to slow DKD progression.

## Figures and Tables

**Table 1:** Baseline characteristics of patients prior to the treatment of sitagliptin (*n* = 102).

Characteristics	Number (%)
*Gender*	
Male	49 (48.04)
Female	53 (51.96)

*Age*	
Younger than 60 years	45 (44.12)
60–70 years	39 (38.24)
Older than 70 years	18 (17.65)

*Body mass index (BMI)*	
Less than 23.0 kg/m^2^	25 (24.51)
23.0–25.0 kg/m^2^	13 (12.75)
More than 25.0 kg/m^2^	62 (60.78)

*Smoking status*	
Nonsmoker	43 (42.16)
Smoker	59 (57.84)

*Comorbidity disease* ^∗^	
Hypertension	58 (56.86)
Dyslipidaemia	58 (56.86)
Stroke	6 (5.88)
Gout	4 (3.92)
Osteoarthritis	3 (2.94)
Cancer	3 (2.94)
Glaucoma	3 (2.94)

*Concomitant medications* ^∗^	
Biguanides	89 (87.25)
HMG-CoA reductase inhibitors	79 (77.45)
Sulfonylureas	42 (41.18)
Calcium channel blockers (CCBs)	33 (32.35)
Angiotensin receptor blockers (ARBs)	31 (30.39)
Aspirin	24 (23.53)
Insulin	19 (18.63)
Angiotensin-converting enzyme inhibitors (ACEis)	17 (16.67)
*β*-Blockers	12 (11.76)
Vitamins	12 (11.76)
Thiazolidinediones	10 (9.80)
Others	1 (0.98)

*Type 2 diabetes mellitus duration*	
≤10 years	54 (52.94)
11–20 years	32 (31.37)
21–30 years	12 (11.76)
≥30 years	4 (3.92)

*CVD risk*	
<10%	68 (66.67)
10–<20%	10 (9.80)
20–<30%	12 (11.76)
30–<40%	3 (2.94)
≥40%	9 (8.82)

*Estimated glomerular filtration rate stage*	
(1) ≥90.00	50 (49.02)
(2) 60.00–89.99	38 (37.25)
(3) 45.00–59.99	6 (5.88)
(4) 30.00–44.99	7 (6.86)
(5) 15.00–29.99	1 (0.98)

*Proteinuria*	
Negative	95 (93.14)
Trace	3 (2.94)
1+	4 (3.92)
2+	0 (0.00)
3+	0 (0.00)

*Laboratory value (median (95% CI)*)	
Estimated glomerular filtration rate (ml/min/1.73 m^2^)	90.80 (78.50–92.34)
Serum creatinine (mg/dL)	0.84 (0.77–0.96)
Systolic blood pressure (mmHg)	136.00 (133.88–144.54)
Diastolic blood pressure (mmHg)	78.00 (74.13–81.02)
Fasting blood sugar (mg/dL)	184.00 (172.47–202.22)
Hemoglobin A1c (%)	8.70 (8.11–9.25)
Total cholesterol (mg/dL)	186.00 (169.53–194.39)
Triglyceride (mg/dL)	148.00 (144.16–198.91)
HDL-cholesterol (mg/dL)	51.00 (48.54–56.77)
LDL-cholesterol (mg/dL)	107.00 (103.87–126.81)
Aspartate transaminase (IU)	20.00 (19.56–37.38)
Alanine transferase (IU)	21.00 (21.13–41.09)

^∗^Each patient could have more than one disease. HDL, high-density lipoprotein; LDL, low-density lipoprotein.

**Table 2:** Spearman correlation coefficients (*r*) and *p* values between kidney parameters including serum creatinine (Scr) and estimated glomerular filtration rate (eGFR) change at 6-month, 12-month, and 18-month posttreatment and factors.

	Kidney parameters' change		
	6 months	12 months	18 months
Age	Scr: *r* = 0.033	Scr: *r* = 0.136	Scr: *r* = 0.432
	*p* value = 0.750	*p* value = 0.297	*p* value = 0.010
	eGFR: *r* = −0.040	eGFR: *r* = −0.132	eGFR: *r* = −0.430
	*p* value = 0.699	*p* value = 0.309	*p* value = 0.010

Body mass index	Scr: *r* = 0.040	Scr: *r* = −0.150	Scr: *r* = 0.053
	*p* value = 0.705	*p* value = 0.247	*p* value = 0.762
	eGFR: *r* = 0.021	eGFR: *r* = 0.142	eGFR: *r* = −0.009
	*p* value = 0.841	*p* value = 0.276	*p* value = 0.961

Diabetes mellitus duration	Scr: *r* = −0.011	Scr: *r* = −0.147	Scr: *r* = 0.284
	*p* value = 0.919	*p* value = 0.264	*p* value = 0.098
	eGFR: *r* = 0.005	eGFR: *r* = 0.195	eGFR: *r* = −0.256
	*p* value = 0.963	*p* value = 0.135	*p* value = 0.138

Baseline fasting blood sugar	Scr: *r* = 0.210	Scr: *r* = −0.080	Scr: *r* = −0.129
	*p* value = 0.042	*p* value = 0.511	*p* value = 0.461
	eGFR: *r* = −0.245	eGFR: *r* = 0.046	eGFR: *r* = 0.081
	*p* value = 0.017	*p* value = 0.727	*p* value = 0.645

Baseline hemoglobin A1c	Scr: *r* = 0.291	Scr: *r* = −0.380	Scr: *r* = −0.265
	*p* value = 0.005	*p* value = 0.772	*p* value = 0.124
	eGFR: *r* = −0.277	eGFR: *r* = 0.000	eGFR: *r* = 0.273
	*p* value = 0.007	*p* value = 0.999	*p* value = 0.112

Baseline total cholesterol	Scr: *r* = 0.128	Scr: *r* = 0.013	Scr: *r* = 0.358
	*p* value = 0.376	*p* value = 0.942	*p* value = 0.145
	eGFR: *r* = −0.108	eGFR: *r* = −0.054	eGFR: *r* = −0.307
	*p* value = 0.454	*p* value = 0.762	*p* value = 0.215

Baseline triglyceride	Scr: *r* = 0.202	Scr: *r* = 0.030	Scr: *r* = 0.534
	*p* value = 0.159	*p* value = 0.868	*p* value = 0.022
	eGFR: *r* = −0.177	eGFR: *r* = −0.018	eGFR: *r* = −0.410
	*p* value = 0.219	*p* value = 0.921	*p* value = 0.091

Baseline HDL-cholesterol	Scr: *r* = −0.168	Scr: *r* = −0.236	Scr: *r* = −0.480
	*p* value = 0.243	*p* value = 0.178	*p* value = 0.044
	eGFR: *r* = 0.158	eGFR: *r* = 0.172	eGFR: *r* = 0.441
	*p* value = 0.274	*p* value = 0.331	*p* value = 0.067

Baseline LDL-cholesterol	Scr: *r* = 0.103	Scr: *r* = 0.010	Scr: *r* = −0.166
	*p* value = 0.329	*p* value = 0.940	*p* value = 0.340
	eGFR: *r* = −0.078	eGFR: *r* = −0.043	eGFR: *r* = 0.238
	*p* value = 0.455	*p* value = 0.740	*p* value = 0.169

Baseline eGFR	Scr: *r* = 0.073	Scr: *r* = −0.003	Scr: *r* = −0.308
	*p* value = 0.486	*p* value = 0.981	*p* value = 0.072
	eGFR: *r* = −0.092	eGFR: *r* = −0.063	eGFR: *r* = 0.219
	*p* value = 0.379	*p* value = 0.629	*p* value = 0.207

Baseline Scr	Scr: *r* = −0.026	Scr: *r* = 0.141	Scr: *r* = 0.254
	*p* value = 0.807	*p* value = 0.280	*p* value = 0.141
	eGFR: *r* = 0.039	eGFR: *r* = −0.069	eGFR: *r* = −0.180
	*p* value = 0.710	*p* value = 0.596	*p* value = 0.300

Baseline aspartate transaminase	Scr: *r* = −0.064	Scr: *r* = 0.238	Scr: *r* = −0.108
	*p* value = 0.685	*p* value = 0.223	*p* value = 0.702
	eGFR: *r* = 0.044	eGFR: *r* = −0.230	eGFR: *r* = 0.170
	*p* value = 0.779	*p* value = 0.230	*p* value = 0.544

Baseline alanine transferase	Scr: *r* = 0.020	Scr: *r* = 0.024	Scr: *r* = 0.056
	*p* value = 0.900	*p* value = 0.905	*p* value = 0.844
	eGFR: *r* = −0.050	eGFR: *r* = −0.021	eGFR: *r* = −0.011
	*p* value = 0.749	*p* value = 0.915	*p* value = 0.970

Statistical significance was set as *p* value <0.05. HDL, high-density lipoprotein; LDL, low-density lipoprotein; eGFR, estimated glomerular filtration rate; Scr, serum creatinine.

## Data Availability

The data that support the findings of this study are available from the corresponding author upon reasonable request.
